# Multidimensional Loneliness Scale: Development and Psychometric Properties of a Peruvian Version

**DOI:** 10.3390/healthcare13151797

**Published:** 2025-07-24

**Authors:** Carlos Pérez-Lara, Melissa Hospinal-Zavaleta, Militza Novoa-Seminario, Mario Sandoval-Rosas, Jesús Saldaña-Bocanegra, Lucy Máximo-Sandoval, Liliana Haro-León, Miguel Benites-Romero, Guicela Cabrejo-Paredes, Doris Lara-Malca

**Affiliations:** 1 Program for Formative Research, Cesar Vallejo University, Trujillo 13001, Peru; dhospinalz@ucvvirtual.edu.pe (M.H.-Z.); militza_06082010@hotmail.es (M.N.-S.); jsaldana@ucv.edu.pe (J.S.-B.); lmaximo@ucv.edu.pe (L.M.-S.); lharol@ucv.edu.pe (L.H.-L.); abenitesr@ucvvirtual.edu.pe (M.B.-R.); gcabrejop@ucvvirtual.edu.pe (G.C.-P.); dlara@ucv.edu.pe (D.L.-M.); 2 Secondary Education Program, Public Higher Pedagogical Education School “Piura”, Piura 20004, Peru; malusaro_23@hotmail.com

**Keywords:** loneliness, isolation, social disconnection, family estrangement, loss of attachment figure, intrapersonal emptiness

## Abstract

**Background/Objectives**: Loneliness is the sensation of feeling alone or emotionally isolated, even when one is surrounded by other people. It is associated with conditions such as depression, anxiety, harmful habits, and cardiovascular problems. The main objective of the present study was to develop and determine the psychometric properties of the Multidimensional Loneliness Scale (MLS), which is a self-report instrument. **Methods:** The present study is instrumental in nature, as it aims to analyze the psychometric properties of a new assessment instrument. A total of 484 adults, both men and women, aged between 18 and 55 years, participated in this research. **Results**: Exploratory factor analysis revealed the presence of four dimensions: social disconnection, family estrangement, loss of attachment figure, and intrapersonal emptiness. Confirmatory factor analysis demonstrated that the four-dimensional model exhibited a good fit (CFI = 0.91; RMSEA = 0.07; SRMR = 0.07; AIC = 737.87). The concurrent validity was evidenced by significant correlations with the De Jong Gierveld scale and the Revised UCLA Loneliness Scale. The reliability analysis demonstrated satisfactory internal consistency, with omega coefficients ranging from 0.84 to 0.92 and alpha coefficients from 0.84 to 0.93. **Conclusions**: The MLS is a self-report instrument designed to assess loneliness, and it has satisfactory psychometric properties.

## 1. Introduction

Loneliness is a phenomenon that profoundly affects many individuals across various communities worldwide, as it involves not only the absence of companionship but also an emotional disconnection that can significantly impact one’s quality of life [1,2,3]. It also has repercussions on physical health, being associated with a higher risk of cardiovascular diseases, sleep disorders, immune system dysfunction, and mortality [4,5,6,7,8]. Moreover, it can affect mental health by increasing the risk of depression, anxiety, chronic stress, and even substance abuse [1,9,10]. Individuals experiencing loneliness also tend to avoid social activities and forming meaningful connections [6,11,12].

Numerous studies have been conducted to measure and understand perceived loneliness across different population groups, such as the elderly, adolescents, and individuals with chronic illnesses [1,11,13,14,15,16]. To adequately address this issue, it is crucial to have instruments that accurately and comprehensively measure the experience of loneliness in its various dimensions.

Currently, several tools exist for measuring loneliness, among which the UCLA Loneliness Scale and the De Jong Gierveld Loneliness Scale are the most prominent. The UCLA Loneliness Scale, developed by Russell et al. (1980) [17], is widely used and treats loneliness as a unidimensional construct, primarily assessing the perception of social and emotional isolation. This scale comprises 20 items and has demonstrated good reliability and validity across diverse populations [18,19,20]. On the other hand, the De Jong Gierveld Loneliness Scale measures both emotional and social loneliness and consists of 11 items [20,21,22,23,24]. Unlike the UCLA scale, this tool divides loneliness into two components: emotional loneliness and social loneliness [23,25,26,27,28]. However, both instruments consider loneliness in general terms and do not delve into other aspects that may influence the experience of loneliness, such as family contexts, the loss of attachment figures, or intrapersonal emptiness.

To address these limitations, the Multidimensional Loneliness Scale (MLS) was developed. Grounded in a biopsychosocial framework, the MLS integrates both individual and contextual factors to more accurately capture the complexity of loneliness across diverse settings and populations [6,29,30,31,32,33]. By including dimensions related to family estrangement and internal emptiness, the MLS identifies subtle facets of loneliness that unidimensional measures fail to detect, thereby supporting more targeted and effective interventions for individuals experiencing this condition.

The theoretical foundation of the MLS is eclectic, drawing upon a multidimensional and biopsychosocial perspective that integrates both individual and contextual factors in the study and measurement of loneliness. This integrative approach is further strengthened by incorporating core concepts from several established psychological theories. Attachment Theory, proposed by Bowlby (1988) [29], emphasizes the importance of early emotional bonds and their impact on one’s ability to form healthy relationships throughout life. Self-Determination Theory, developed by Ryan and Deci (2000) [31], highlights the role of intrinsic motivation and the fulfillment of psychological needs in fostering well-being and social connectedness. From a cognitive behavioral perspective, Beck (1976) [32], provides insights into how cognitive processes and behavioral patterns influence emotional experiences and social interactions. This theoretical integration reinforces the conceptual foundation of the MLS and supports its applicability across diverse contexts.

For the development of the MLS, the following definition of loneliness was formulated: Loneliness is the feeling of being alone or emotionally isolated, even when in the presence of others. It is a subjective experience characterized by a perceived lack of meaningful connection with others or a persistent sense of isolation, even when physically accompanied. Loneliness does not necessarily depend on the quantity of social interactions, but rather on the quality of those interactions and the extent to which they fulfill emotional needs for connection and support [6,8]. Based on this conceptualization, four core dimensions of loneliness were identified: social disconnection, family estrangement, loss of attachment figures, and intrapersonal emptiness.

To improve the clarity and readability of the theoretical framework, the four dimensions of loneliness proposed in this study are presented in Table 1. These dimensions were developed based on a comprehensive review of the literature and provide the conceptual foundation for the Multidimensional Loneliness Scale.

This research is justified by the need for an instrument capable of assessing perceived loneliness across multiple dimensions. This approach allows for the early detection and addressing of the problem, providing timely interventions and promoting strategies to enhance the emotional and social connection of those experiencing loneliness. This, in turn, can help prevent physical and mental health complications and improve the quality of life and social integration of individuals facing perceived loneliness. Proper evaluation of loneliness also enables researchers to study its causes and consequences and develop more effective interventions to address this significant public health issue.

This study aims to develop and evaluate the psychometric properties of the Multidimensional Loneliness Scale (MLS) in an adult population, following the standards of AERA, APA, and NCME [40]. The specific objectives were as follows:

(1) Conceptualize, identify, and determine MLS items.

(2) Analyze psychometric properties, including content validity through expert judgment, construct validity through factor analysis, and concurrent criterion validity, in the adult population.

(3) Determine the psychometric property of reliability through internal consistency.

## 2. Method

### 2.1. Study Design

This instrumental study aimed to develop a multidimensional loneliness scale with adequate psychometric properties intended for Peruvian adults [41].

### 2.2. Procedure and Participants

**Figure 1:**
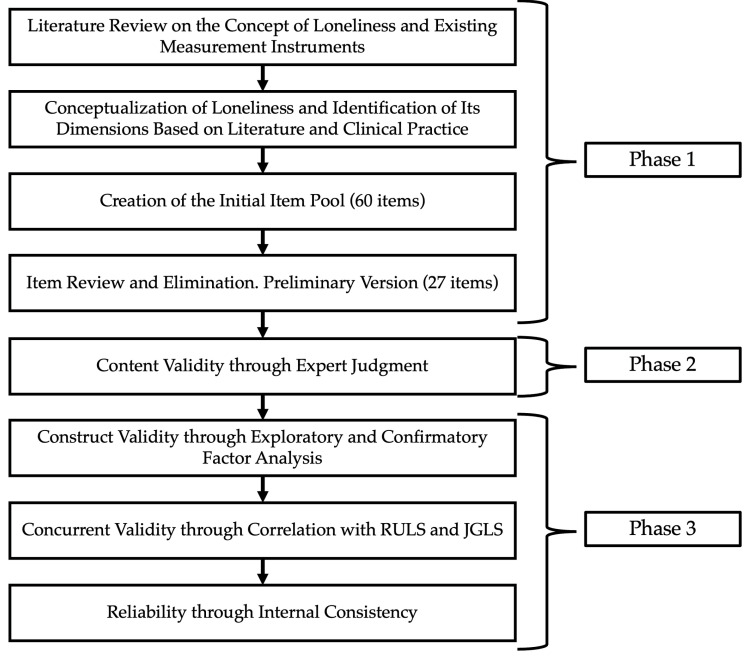
Workflow of the development and psychometric properties of the MLS.

The study was conducted in three sequential phases, as illustrated in Figure 1.

(a) First Phase: Development of the MLS Items

In the first phase, a preliminary version of the scale was developed based on the researchers’ clinical experience, an exhaustive review of the scientific literature, and the analysis of previously developed instruments for assessing loneliness. Drawing from this information, a conceptual definition of the construct “loneliness” and its theoretical dimensions were established, which guided the construction of the initial items of the instrument. The initial item pool consisted of 60 questions, which were developed by half of the research team. This initial version contained more than twice the number of final items, as recommended by Kline (2013) [42,43]. Subsequently, the items were reviewed and refined by the other half, eliminating similar items, those containing multiple ideas, as well as those that were lengthy or ambiguous. This process resulted in a preliminary version of the instrument with 27 items, which was then subjected to expert evaluation.

(b) Second Phase: Content Validity of the MLS Items

In the second phase, content validity was assessed using the expert judgment method. Seven experts participated in this procedure in accordance with the criteria recommended by the American Psychological Association and the Educational Research Association [40,44]. The inclusion criteria required that participants be mental health professionals, either psychologists or psychiatrists, with a master’s or doctoral degree, and a minimum of five years of experience in providing care to patients in individual, family, and group therapy settings. The experts evaluated each item in a single round, assessing its clarity, coherence, and relevance. The instrument adopted a Likert-type response format, where participants had to choose one of the four available response options: 1 = “never”, 2 = “sometimes”, 3 = “almost always”, or 4 = “always”. The score for each dimension is obtained by summing the scores assigned to the items that compose it.

(c) Third Phase: Assessment of Psychometric Properties

In the third phase, the psychometric properties of the scale were examined. Evidence of construct validity and concurrent validity was assessed, along with the reliability of the instrument. This stage involved the participation of 484 Peruvian adults, predominantly women (70.5%), with an age range of 18 to 55 years (M age = 28.8 years; SD = 11.2). Inclusion criteria required participants to be men or women within the specified age range, from diverse sociodemographic backgrounds, with intact cognitive functioning, and with access to social support networks. Exclusion criteria encompassed incomplete responses, the loss of a family member within the past year, or a prior psychological or psychiatric diagnosis of mood disorders. Additionally, individuals reporting excessive substance use or severe chronic illnesses were excluded in order to minimize confounding variables that could affect the perception of loneliness and compromise the validity of the findings.

The total sample was randomly divided into two groups of 242 participants each. The EFA group, composed of individuals aged 18 to 55, had a mean age of 29.4 years (SD = 11.0), while the CFA group, within the same age range, had a mean age of 27.8 years (SD = 10.9). The similarity in age distribution across both groups supports their comparability and appropriateness for the respective analyses. The procedure for sample division, as well as the chosen subgroup sizes, is well supported by the literature [45,46,47]. Table 2 presents the sociodemographic characteristics of the participants.

Participant recruitment was conducted using the snowball sampling technique and social media dissemination. A single Google Forms link was used, which was distributed by the research team members via WhatsApp and posts in Facebook groups. Initially, the researchers shared the link with their personal and professional contacts, who, in turn, forwarded it to their acquaintances, allowing for a gradual expansion of participant recruitment. The link was posted in general-interest Facebook groups, while groups specifically related to mental health were deliberately avoided. This decision aimed to reduce potential biases and minimize the impact of social desirability on responses. In cases where group administrators required prior approval for posting, permission was requested, and the moderators were informed about the study’s purpose. However, in groups where open posting was allowed, the link was shared without prior contact with administrators. It is important to note that participation in the study was entirely voluntary, and no incentives or financial compensation were offered to participants.

Before answering the questions, participants were instructed to complete the questionnaires at their own pace and in a distraction-free environment, with an estimated completion time of approximately 20 min. Informed consent was obtained electronically, ensuring anonymity and confidentiality. Participants then completed the Multidimensional Loneliness Scale (MLS), the De Jong Gierveld Loneliness Scale, and the Revised UCLA Loneliness Scale. All instruments were administered via the Google Forms platform and accessed through a single link.

After completing the instruments, the process for handling invalid questionnaires involved a thorough review of response quality. Questionnaires exhibiting issues such as incomplete answers or irregular response patterns (e.g., selecting the same option across all items) were considered for exclusion. However, no questionnaires met the criteria for invalidity, and thus, none were removed. Following this quality control procedure, all 484 questionnaires were deemed valid and subsequently included in the analysis.

### 2.3. Measures

#### 2.3.1. Multidimensional Loneliness Scale (MLS)

The Multidimensional Loneliness Scale (MLS) is a self-report instrument in the Spanish language consisting of 25 items designed to assess individuals’ perceived level of loneliness. Items are rated on a 4-point Likert scale ranging from 1 (never) to 4 (always). This scale was developed to measure four dimensions: social disconnection, family distancing, loss of attachment figures, and intrapersonal emptiness. Scores are calculated by summing the points of the items within each dimension, yielding individual scores for each. A higher score indicates a greater presence of loneliness and its respective dimensions. The scale demonstrated excellent levels of internal consistency (for a more detailed factorial structure and reliability, refer to the Results section).

#### 2.3.2. De Jong Gierveld Loneliness Scale

The De Jong Gierveld Loneliness Scale is a self-report instrument consisting of 11 items used to assess loneliness as a unidimensional variable, with response options of 1 = “no”, 2 = “more or less”, and 3 = “yes”. The original author of the instrument suggested dichotomizing items by assigning a point to “more or less” or “no” responses for negative items (1, 4, 7, and 11) or a point to “more or less” or “yes” responses for positive items (2, 3, 5, 8, 9, and 10). With this adjustment, the scale ranges from 0 to 11 points, where a higher score indicates a greater level of loneliness. In the study sample, an adequate level of reliability was achieved (KR20 = 0.826) [21].

#### 2.3.3. UCLA Loneliness Scale Revised

The revised UCLA Loneliness Scale is a self-reported scale consisting of 20 items that assess loneliness in diverse populations, treating loneliness as a unidimensional variable. The response options are 1 = “never”, 2 = “rarely”, 3 = “sometimes”, and 4 = “frequently”. The scale score is obtained by summing all points according to the marked responses, with a minimum score of 20 and a maximum of 80, where a higher score indicates higher loneliness levels. In the study sample, the scale demonstrated high internal consistency and reliability (α = 0.91, ω = 0.91) [17].

### 2.4. Statistical Analysis

A descriptive analysis of the items from the Multidimensional Loneliness Scale (MLS) was performed to assess data normality and suitability. Means, variances, skewness, and kurtosis coefficients were calculated for each item using SPSS software (version 27) [48]. Additionally, Mardia’s test of multivariate normality was applied, with *p* values greater than 0.05 interpreted as indicative of a normal distribution. The suitability of the data for EFA was further evaluated using Bartlett’s test of sphericity and the Kaiser–Meyer–Olkin (KMO) measure of sampling adequacy. These analyses were conducted using JASP software (version 0.18.1) [49].

Exploratory factor analysis (EFA) was conducted using a polychoric correlation matrix and the minimum residual (MinRes) extraction method with oblique (Oblimin) rotation, which is appropriate for correlated factors, as it facilitates interpretation and improves model fit. The Kaiser–Meyer–Olkin (KMO) measure and Bartlett’s test of sphericity were used to assess the suitability of the data for factor analysis [46,50]. Items with factor loadings below 0.40 were removed [46]. All EFA analyses were performed using JASP software (version 0.18.1) [49].

The factorial structure was tested using confirmatory factor analysis (CFA), with a good model fit defined by a nonsignificant chi-square (χ2) value (*p* > 0.05), a comparative fit index (CFI) greater than 0.90, and a root mean square error of approximation (RMSEA) and a standardized root mean square residual (SRMR) less than 0.08, and the Akaike information criterion (AIC) was used to compare model fit, with lower AIC values indicating a more parsimonious and preferable model relative to competing models [51,52]. The fits of two factorial structures, (i) a single-factor model and (ii) a four-factor model, were compared. CFA analyses were performed using SPSS (version 27) and AMOS 26.0 [53].

To assess concurrent validity, correlations were obtained between each dimension score of the MLS and the total scores of the Revised UCLA Loneliness Scale and the De Jong Gierveld Loneliness Scale, as these scales are more frequently cited in the literature. These analyses were conducted using SPSS (version 27).

The reliability of the scale was examined using the internal consistency method with Cronbach’s alpha and McDonald’s omega coefficients [54]. Reliability analyses were performed using JASP software (version 0.18.1).

The use of SPSS (version 27) and JASP (version 0.18.1) was based on their reliability, accessibility, and suitability for the statistical procedures required in this study. SPSS was employed for descriptive statistics and correlational analyses due to its robust data handling capabilities and widespread use in psychological research. JASP was selected for exploratory factor analysis and reliability assessments, particularly because it supports polychoric correlation matrices and offers advanced options for psychometric evaluation with an intuitive interface. Confirmatory factor analysis was performed using AMOS (version 26.0), which integrates well with SPSS and allows for precise modeling of latent variables, making it especially appropriate for testing the factorial structure of psychological instruments.

## 3. Results

### 3.1. Content Validity of the MLS

The 27 items of the preliminary version of the MLS were evaluated by experts based on three core criteria: clarity, coherence, and relevance. The scores assigned were analyzed using Aiken’s V statistical test, yielding values ranging from 0.96 to 1.00. None of the experts suggested the inclusion of additional items, indicating adequate comprehensiveness of the instrument. Taken together, these results provide evidence of strong and satisfactory content validity.

### 3.2. Descriptive Analysis of the Items

Table 3 presents the descriptive statistics. Both the absolute skewness and kurtosis values exceeding 1, along with Mardia’s test of multivariate normality, indicate a departure from a normal distribution in the data. Consequently, a polychoric matrix was used for the factor analysis, as this method is more suitable for data with non-normal distributions [46].

### 3.3. Exploratory Factor Analysis

The exploratory factor analysis (EFA) was conducted using a polychoric matrix, and the extraction method involved obtaining the minimum residuals with oblique rotation (Oblimin). A Kaiser–Meyer–Olkin (KMO) index of 0.75 was obtained, along with a statistically significant difference according to Bartlett’s test of sphericity (*p* < 0.001), suggested the appropriateness of the data for factor analysis [46]. The results of the factor loadings are displayed in Table 4.

The exploratory factor analysis (EFA) of the Multidimensional Loneliness Scale (MLS) reveals an underlying structure consisting of four primary factors that account for the variance in participants’ responses. These factors are social Disconnection, family estrangement, loss of attachment figure, and intrapersonal emptiness. Each of these factors represents a distinct dimension of loneliness, and together they explain a significant proportion of the total variance, highlighting the multidimensional nature of this experience.

The first factor, social disconnection, contributes the most to the explained variance, indicating that feelings of not fitting in socially and lacking someone to share with are central components of loneliness. This factor encompasses items that describe how individuals may feel excluded or disconnected from their surroundings, even when they are in the presence of others. However, certain items demonstrated loadings on more than one factor. Items 6 and 7, for instance, showed significant loadings on both the first factor (social disconnection) and the second factor (family estrangement). This finding suggests that the sense of not being valued or understood can manifest both in social relationships and within the family. The overlap of these items underscores the interconnectedness between a lack of social support and the perceived emotional distance within family dynamics. It is possible that some individuals experience loneliness in a similar way across these contexts, reflecting the challenge of distinguishing between social and familial sources of isolation in certain cases.

The second factor, family estrangement, groups items that express feelings of detachment from family members. Individuals may feel as though they are not part of their family, are not understood, or that others are too preoccupied to pay attention to them. This complements social disconnection, suggesting that loneliness can be deeply felt within the family unit.

The third factor, loss of attachment figure, pertains to the longing for a significant person who is no longer present in the individual’s life. Items such as “I think about an important person to me who is no longer with me” reflect a specific type of loneliness associated with the loss of significant relationships, emphasizing the emotional impact of separation or loss.

Finally, the fourth factor, intrapersonal emptiness, includes items that describe behaviors individuals adopt to avoid feeling lonely, such as staying busy or resorting to distractions. This factor appears to capture a more introspective aspect of loneliness related to avoidance and the internal sense of emptiness.

### 3.4. Confirmatory Factor Analysis

**Figure 2:**
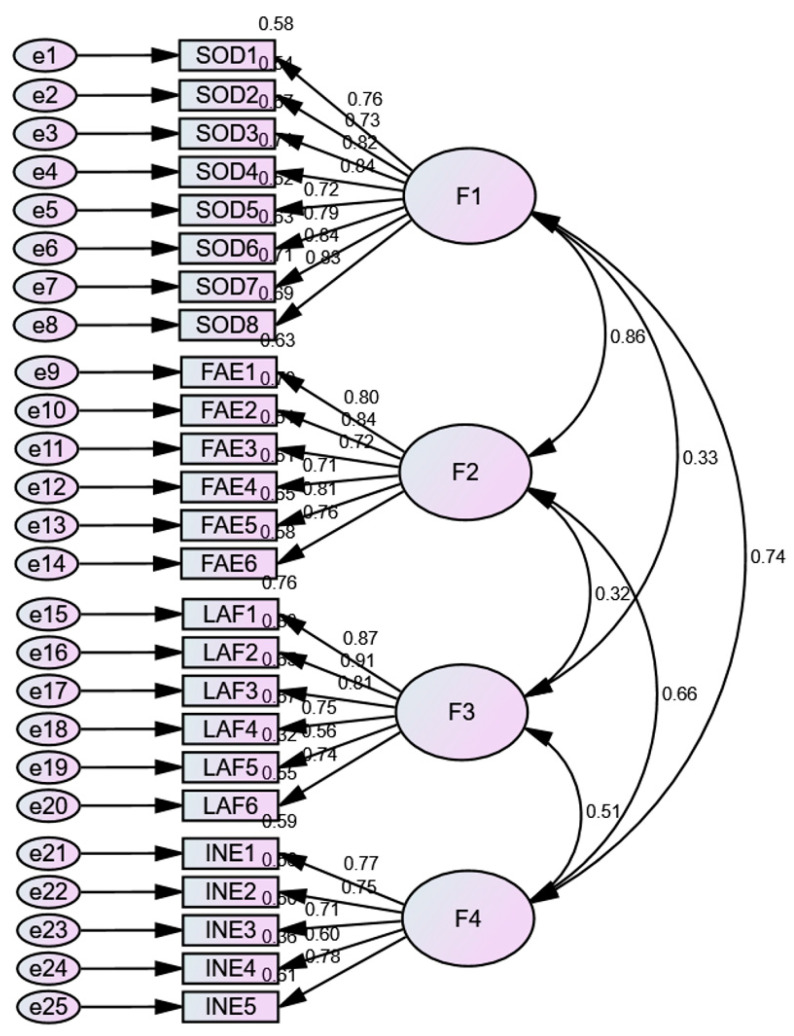
Graphical representation of the factorial structure model for the MLS.

For the confirmatory factor analysis (CFA), two models were examined: one comprising a single factor and the other incorporating the four theorized dimensions. Although both models yielded significant chi-square test results, additional data indicate a favorable fit for the four-dimensional model (CFI = 0.91; RMSEA = 0.07; SRMR = 0.72; AIC = 1730.41). Conversely, the single-factor model exhibited a poor fit (CFI = 0.67; RMSEA = 0.14; SRMR = 0.11; AIC = 737.87), as shown in Table 5. Furthermore, the structural equation model diagram is illustrated in Figure 2.

Figure 2 presents the graphical representation of the factorial structure model for the Multidimensional Loneliness Scale (MLS). The model identifies four main factors: social disconnection (F1), family estrangement (F2), loss of attachment figure (F3), and intrapersonal emptiness (F4). Each of these factors is represented by its corresponding items (SOD, FAE, LAF, and INE), with their respective factor loadings indicated by unidirectional arrows. Correlations between the factors are indicated by numerical values alongside the bidirectional arrows, suggesting significant relationships between the different components of loneliness in the evaluated sample. This model supports the multidimensional nature of loneliness identified in the study.

### 3.5. Concurrent Validity

Table 6 presents the results of the concurrent validity analysis between the Multidimensional Loneliness Scale (MLS) and two well-established loneliness scales: the De Jong Gierveld Loneliness Scale and the Revised UCLA Loneliness Scale. These correlations, assessed using Spearman’s test, help determine the extent to which the MLS dimensions align with the constructs measured by these established scales.

The correlations between the De Jong Gierveld Loneliness Scale and the MLS dimensions are significant across the board (*p* < 0.01). The dimensions of social disconnection (SOD) and family alienation (FAE) show the strongest correlations, with coefficients of 0.65 and 0.59, respectively. This indicates a strong relationship between the loneliness measured by this scale and the social and familial isolation captured by the MLS. Regarding the emotional loneliness subscale, the correlations are more moderate but still significant, with social disconnection (SOD) showing a correlation of 0.44. This suggests that emotional loneliness is related to social disconnection. On the other hand, the social loneliness subscale shows higher correlations, particularly with social disconnection (0.65) and family alienation (0.59), indicating a strong association between these dimensions.

The Revised UCLA Loneliness Scale also shows significant correlations with the MLS dimensions. The highest correlations were found with the social disconnection (SOD) dimension, with a coefficient of 0.75, indicating a very strong relationship between this scale and the perception of social disconnection. The other dimensions also show significant, though lower, correlations, with family alienation (0.61) standing out. Conversely, the attachment figure loss (LAF) dimension shows the lowest correlation (0.20), suggesting that this specific dimension of loneliness is less strongly related to the aspects measured by the UCLA scale.

The Multidimensional Loneliness Scale demonstrates considerable concurrent validity with the scales used, particularly in the dimensions of social disconnection and family alienation. The lower correlations in the attachment figure loss dimension suggest that this dimension may capture a more specific aspect of loneliness that is not fully reflected in the other scales. These findings support the use of the MLS as a valid tool for assessing loneliness in its multiple facets.

### 3.6. Reliability

The reliability results of the Multidimensional Loneliness Scale (MLS) indicate that the four dimensions it comprises exhibit excellent internal consistency. The social disconnection factor obtained an omega coefficient of 0.93 and an alpha of 0.93, while the family estrangement factor showed similar values, with an omega of 0.89 and an alpha of 0.89. The loss of attachment figure factor also reflected high levels of reliability, with an omega of 0.89 and an alpha of 0.90. Finally, the intrapersonal emptiness factor reported omega and alpha coefficients of 0.84. These results confirm that the MLS is a reliable tool for assessing various dimensions of loneliness [54] (see Table 7).

### 3.7. Conversion of Raw Scores to Percentiles

The Table 8 presents the conversion of raw scores to percentiles for the dimensions assessed by the scale: social disconnection, family estrangement, loss of attachment figure, and intrapersonal emptiness. The conversion of raw scores to percentiles was carried out in accordance with recommendations established in the psychometric literature [40,55], which emphasize that percentile scores provide an intuitive interpretation of individual performance relative to a normative sample. To ensure precision and comparability, percentiles from 1 to 99 were calculated based on the empirical distribution of the data using the ordinal method with nearest-rank interpolation [56]. This approach enables each raw score to be linked to its relative position within the sample, offering greater fidelity and sensitivity compared to classifications based on broad ranges or irregular percentiles. In addition, a summary conversion table in five-point percentile intervals is presented, which preserves technical rigor and facilitates clinical interpretation, in accordance with common practices in test standardization [52,55]. Based on these values, cutoff points are established to classify levels as low, medium, and high. Scores corresponding to the 25th percentile or below are categorized as low, scores between the 34th and 67th percentiles are classified as medium, and those above the 75th percentile are considered high [57,58]. This classification allows for precise interpretation of results and facilitates the identification of cases according to severity in each dimension, providing a valuable framework for clinical and research analysis.

## 4. Discussion

The identification of loneliness in individuals is crucial from a health care perspective, as chronic loneliness has profound implications for emotional well-being, physical health, and social interactions [6,11,12,59]. This condition is associated with elevated levels of anxiety, depression, and stress [1,9,10], as well as an increased risk of cardiovascular problems, hypertension, obesity, and immune system dysfunctions [4,5,6,7,8]. Simultaneously, loneliness undermines one’s quality of life by affecting one’s sense of belonging and life satisfaction, influencing both the individual and society as a whole. The early detection of loneliness allows for the implementation of intervention and support strategies, contributing to reducing potential negative consequences and fostering a more connected and healthy society.

Various instruments currently exist for measuring loneliness, such as the UCLA Loneliness Scale (RULS) [17,18,19] and the De Jong Gierveld Loneliness Scale [21,22,23]. However, these instruments consider loneliness to be a unidimensional construct, whereas subsequent studies have identified two dimensions: perceptions of loneliness and satisfaction with social relationships [20].

Therefore, the main objective of the present study was to design and evaluate the psychometric properties of the Multidimensional Loneliness Scale (MLS), which is a novel tool aimed at identifying loneliness through the assessment of four dimensions, namely, social, familial, affective bond, and intrapersonal aspects. The formulation of the MLS and the item generation process were based on current theoretical considerations supported by previous research on loneliness and its dimensions [6,8,34,38,60]. The MLS is presented as a self-assessment instrument designed to address perceived loneliness and its various facets. This questionnaire was developed to capture the subjective experience of loneliness in a multidimensional context; individuals are allowed to self-assess their own loneliness through four dimensions: social disconnection, family distancing, loss of attachment figures, and intrapersonal emptiness. The inclusion of these dimensions provides a more comprehensive view of the experience of loneliness and enables a more accurate and detailed assessment, contributing to a more holistic understanding of the phenomenon and the early identification of those who could benefit from appropriate interventions and support.

The MLS has suitable psychometric characteristics and is supported by robust indicators of validity and reliability. Exploratory factor analysis (EFA) revealed the existence of four distinct dimensions: social disconnection, family distancing, loss of attachment figures, and intrapersonal emptiness (Table 4). The confirmatory factor analysis (CFA) results affirmed the statistical goodness of fit of the four-dimensional model (refer to Table 5). This model enriches previously used instruments for loneliness assessment, such as the De Jong Gierveld Loneliness Scale and the Revised UCLA Loneliness Scale, by incorporating dimensions for evaluation. This structure reflects the inherent complexity of the psychological construct of loneliness, which can manifest in various contexts, encompassing social, familial, and even personal contexts, whether in situations where the company of an attachment figure is missing or in cases where no apparent cause justifies it. This diversity of manifestations is intrinsically linked to an individual’s subjective perception, implying that loneliness can be a personal experience. However, it is essential to recognize that its subjective nature does not diminish its capacity to generate emotional distress. An individual may experience a profound sense of loneliness despite being surrounded by others. This phenomenon highlights the inherent complexity of this construct and its influence on individuals’ psychological well-being. Understanding these multiple dimensions and their impact on emotional well-being is essential for the effective evaluation of loneliness in the fields of psychology and mental health. The results underscore the statistical superiority of the four-dimensional model compared to the unidimensional model, suggesting that MLS items measure different factors or dimensions of the same construct.

Regarding the concurrent validity of the Multidimensional Loneliness Scale (MLS), the highest correlations were observed when analyzing the total scores of the De Jong Gierveld Loneliness Scale (JGS) [22,23] and the Revised UCLA Loneliness Scale (RULS) [17]. The correlation values for these global scales ranged from 0.65 to 0.75 (*p* < 0.01), indicating a strong and statistically significant relationship between the MLS dimensions and both scales. However, a low correlation of 0.20 (*p* < 0.01) was found between the “loss of attachment figure” dimension and the total RULS score. This result suggests that, while the MLS generally demonstrates strong concurrent validity, some specific dimensions, particularly the “loss of attachment figure”, show a low relationship with general loneliness measures used in other scales.

It is important to note that this low correlation may stem from the fact that the “loss of attachment figure” dimension captures a specific aspect of loneliness that is not fully considered by scales such as the JGS and RULS. While these scales tend to assess loneliness from a broader perspective, focusing on general emotional and social disconnection, the “loss of attachment figure” dimension of the MLS addresses a more intimate and specific form of loneliness related to the absence of significant attachment figures. This suggests that the MLS captures a deeper and more personal experience of loneliness, not fully reflected in traditional instruments, which may explain the low correlation in this dimension.

On the other hand, the MLS dimensions showed significant correlations with the “emotional loneliness” and “social loneliness” dimensions, as proposed in later studies [20]. When analyzing the specific subscales of the JGS, such as “social disconnection” and “emotional loneliness”, higher correlations were observed. The “social disconnection” dimension showed values between 0.44 and 0.75 (*p* < 0.01), indicating a strong relationship with scales measuring social loneliness. In contrast, the correlation between the “loss of attachment figure” and the “emotional loneliness” dimension was only 0.14 (*p* < 0.05). This suggests that, although the MLS adequately reflects different forms of loneliness, emotional loneliness related to the loss of attachment figures is a more specific dimension that is not fully captured by other scales, reinforcing the added value of the MLS in the multidimensional assessment of loneliness.

These findings lay the groundwork for future research that can expand our understanding of this phenomenon. Furthermore, the importance of having an instrument that measures perceived loneliness across four dimensions lies in its ability to provide a more comprehensive and detailed understanding of the loneliness experience. Each of these dimensions reflects various aspects of loneliness, ranging from social disconnection and familial distancing to the loss of attachment figures and the experience of intrapersonal emptiness. This multidimensional approach allows for a more precise assessment of the nature and severity of loneliness in individuals, facilitating the design of targeted interventions and support strategies to address problematic areas. Ultimately, the detailed measurement of these dimensions contributes to a better understanding of the causes and implications of loneliness, benefiting individual care and emotional well-being while also advancing scientific research in this field.

### 4.1. Limitations

Several considerations should be noted regarding the inherent limitations of this study and avenues for future research in this field. First, the use of snowball sampling may have introduced certain biases due to its nonrandom nature. Future research could employ random sampling methods to minimize this potential bias. Additionally, the composition and size of the sample, although meeting the minimum requirements for robust statistical analyses such as EFA and CFA, may have introduced bias into the findings. The sample was predominantly composed of women, which could limit the generalizability of the results to more diverse populations. To address this limitation, future research should consider using larger and more gender-balanced samples to enhance the representativeness of the findings.

Another limitation relates to the exclusive use of self-reports, which may have introduced response biases, such as social desirability bias, potentially influencing responses related to family and social aspects [61,62]. Although solid evidence of concurrent validity was obtained, the lack of a specific assessment of divergent validity represents a limitation. Future studies should consider this evaluation, particularly in mental health contexts, to ensure an adequate distinction between loneliness and other constructs such as depression or anxiety.

Finally, evidence of multidimensionality was observed in both the exploratory and confirmatory factor analyses, suggesting that the scale may not be fully represented by a single total score. Without testing bifactor or second-order factor models, it is not possible to confirm the validity of a total score that encompasses all dimensions. Future research could explore these models to ensure the appropriateness of a total score for this multidimensional construct.

### 4.2. Implications for Practice

The construction and psychometric properties of the Multidimensional Loneliness Scale (MLS) have significant implications for various domains, particularly mental health. The development of the MLS provides a specific and validated tool for assessing loneliness in adults, contributing to a more precise and reliable measurement of this phenomenon. The MLS allows for the identification of specific areas—social disconnection, family distancing, loss of attachment figures, or intrapersonal emptiness—where loneliness is most prevalent. Early detection using the MLS can help identify psychological issues related to loneliness at an early stage and pinpoint the affected dimension. Recognizing signs of perceived loneliness in specific areas can facilitate early and preventive interventions, enabling more effective therapeutic approaches tailored to the affected dimension. This can help adults avoid the development of more severe mental health problems [63,64,65]. Thus, the inclusion of the MLS in mental health assessments allows for a more comprehensive approach to addressing emotional issues.

Loneliness has ramifications for both mental and physical health, and the MLS offers a tool to assess the relationship between loneliness and other health aspects, contributing to comprehensive intervention strategies. Its implementation in routine assessments can enhance the identification of adults at risk of psychological problems associated with loneliness, enabling earlier and more specific attention [9,14,59]. Understanding perceived loneliness through the MLS can support the design of more effective intervention strategies. This includes the implementation of social support programs, specific therapies, and other interventions aimed at addressing both the emotional and physical aspects associated with loneliness.

In the educational domain, the MLS emerges as a valuable tool for adult learners, linking variables to academic performance, identifying issues, and proposing strategies to improve learning quality and prevent academic failure. In the workplace, perceived loneliness among adults negatively affects organizational commitment, productivity, and job satisfaction. The multidimensional nature of the MLS offers the possibility of evaluating workplace loneliness from various perspectives, enabling organizations to identify specific profiles and design personalized interventions to improve employee well-being and performance.

## 5. Conclusions

The Multidimensional Loneliness Scale (MLS) was developed to assess the perception of loneliness in adults through a multidimensional lens. The construction of the instrument was based on current theoretical frameworks, allowing for the conceptualization of four key dimensions and the formulation of items that reflect the complexity of this experience. The content validity of the items was confirmed through expert judgment, while the construct validity was supported by both exploratory and confirmatory factor analyses, which consistently revealed a four-factor structure. Additionally, concurrent validity analyses demonstrated significant associations with established measures of loneliness. The reliability of the instrument was confirmed through high internal consistency coefficients across all dimensions. These results indicate that the MLS is a psychometrically sound instrument that is suitable for evaluating different facets of loneliness in the adult population.

## Figures and Tables

**Table 1:** Theoretical dimensions of loneliness proposed in the present study.

Dimension	Definition
Social Disconnection	This dimension addresses the subjective perception of a lack of connection and belonging with others. It represents an emotional experience in which an individual may experience the absence of meaningful relationships or a lack of integration into their social environment. Despite being surrounded by people, the feeling of social disconnection can lead to loneliness, misunderstanding, or neglect, contributing to a sense of isolation. Various factors, such as a shortage of meaningful interactions, low self-esteem, difficulties in social skills, significant life changes, geographic isolation, and the absence of support groups, can contribute to this perception. Social disconnection is not necessarily linked to the quantity of interactions but rather to the quality and satisfaction of these relationships. Even in seemingly active environments, a lack of deep relationships can generate a sense of disconnection and loneliness [4,6,28,33,34,35].
Family Estrangement	This dimension describes the emotional experience in which an individual feels undervalued, misunderstood, or inadequately included within their family. Even if satisfying relationships are maintained outside the family nucleus, disconnection from close relatives can lead to loneliness and a lack of support within the home. Factors such as unresolved conflicts, lack of effective communication, differences in values or interests, emotional neglect, and traumatic situations can contribute to family estrangement. This lack of emotional connection can significantly impact emotional well-being, manifesting as feelings of sadness, anxiety, and low self-esteem. It is relevant to note that the intensity and duration of estrangement can vary among individuals and specific family dynamics [6,33,34,36].
Loss of Attachment Figure	This dimension refers to the subjective experience of feeling emotionally separated from a significant figure due to loss, whether by death, separation, or breakup. Loss can lead to a perception of painful emotional separation that is challenging to cope with. This sensation can manifest as emptiness, loneliness, or deep sadness. The intensity and duration of this perception vary among individuals and according to the circumstances of the loss. Some may adapt gradually, while others may need time and support to heal emotionally [30,36,37,38].
Intrapersonal Emptiness	This dimension pertains to the emotional experience in which a person feels loneliness or emptiness without a clearly identifiable cause. It involves a general sense of emotional discontent, dissatisfaction, or a lack of purpose in life, without being able to attribute it to a specific circumstance or the absence of an attachment figure. This experience may lead individuals to seek distractions or superficial connections to avoid directly confronting the underlying cause of their feelings. This indicates the need to explore deeper emotional and psychological aspects, such as the search for purpose, work on self-esteem and self-acceptance, or the development of more meaningful relationships [34,35,39].

Note. The definitions presented here are derived from the theoretical framework developed by the authors based on the existing literature.

**Table 2:** Sociodemographic Characteristics of Participants.

Characteristics		EFA		CFA		Total	
		*n*	%	*n*	%	*n*	%
Gender							
	Female	164	67.8%	177	73.1%	341	70.5%
	Male	78	32.2%	65	26.9%	143	29.5%
Marital Status							
	Single	141	58.3%	182	75.2%	323	66.7%
	Married or cohabiting	87	36.0%	43	17.8%	130	26.9%
	Separated	14	5.8%	17	7.0%	31	6.4%
Children							
	Without children	141	58.3%	184	76.0%	325	67.1%
	With children	101	41.7%	58	24.0%	159	32.9%

Note. *N* = 484 (EFA group *n* = 242; CFA group *n* = 242). Participants had a mean age of 28.8 years (SD = 11.2), and age did not differ between the EFA and CFA groups.

**Table 3:** Descriptive statistics of the items of the Multidimensional Loneliness Scale (MLS).

Item	Mean	95% CI	Variance	Skewness	Kurtosis	Uniqueness
1	2.38	(2.22–2.55)	0.96	0.35	−0.78	0.23
2	2.37	(2.22–2.51)	0.79	0.47	−0.52	0.27
3	2.03	(1.88–2.18)	0.86	0.72	−0.26	0.33
4	2.10	(1.93–2.27)	1.09	0.61	−0.80	0.38
5	2.03	(1.88–2.17)	0.82	0.66	−0.28	0.52
6	2.03	(1.88–2.17)	0.81	0.70	−0.17	0.46
7	1.70	(1.58–1.82)	0.53	0.91	0.72	0.46
8	1.48	(1.36–1.60)	0.52	1.54	2.07	0.44
9	2.11	(1.95–2.26)	0.90	0.66	−0.39	0.58
10	1.73	(1.59–1.87)	0.72	1.04	0.40	0.48
11	1.67	(1.54–1.80)	0.63	1.15	0.94	0.48
12	1.41	(1.29–1.53)	0.51	1.98	3.74	0.56
13	1.68	(1.56–1.81)	0.56	1.07	1.05	0.35
14	1.58	(1.46–1.71)	0.57	1.32	1.48	0.27
15	1.78	(1.65–1.91)	0.63	0.96	0.73	0.38
16	1.62	(1.49–1.74)	0.59	1.33	1.63	0.33
17	1.51	(1.38–1.64)	0.60	1.63	2.31	0.32
18	1.72	(1.58–1.85)	0.63	1.05	0.75	0.46
19	1.73	(1.59–1.87)	0.70	1.06	0.52	0.34
20	1.86	(1.72–2.00)	0.72	0.77	−0.06	0.45
21	1.90	(1.76–2.04)	0.70	0.66	−0.21	0.37
22	1.74	(1.60–1.89)	0.79	1.06	0.31	0.35
23	2.04	(1.88–2.20)	0.97	0.62	−0.66	0.48
24	1.74	(1.61–1.88)	0.70	1.06	0.60	0.50
25	1.57	(1.43–1.71)	0.70	1.46	1.37	0.44
**Mardia’s Test of Multivariate Normality**			**Value**	**Statistic**	**df**	***p***
Skewness			175.61	7083.01	2925	<0.001
Small Sample Skewness			175.61	7177.63	2925	<0.001
Kurtosis			889.40	45.39		<0.001

Note. *N* = 242. The statistic for skewness is assumed to be chi-square-distributed and the statistic for kurtosis standard normal. The Mardia’s test of multivariate normality was applied to assess the skewness and kurtosis of the data. The results reveal significant skewness (175.61, *p* < 0.001) and kurtosis (889.40, *p* < 0.001), indicating a non-normal distribution.

**Table 4:** Results of exploratory factor analysis for the Multidimensional Loneliness Scale (MLS).

MLS Item		Loading Factor				s	k
		1	2	3	4		
Factor 1: Social Disconnection							
	1. I do not have common conversation topics with the people around me.	**0.88**	−0.15	0.02	0.00	0.35	−0.78
	2. I feel like I do not ‘fit in’ with the people around me.	**0.84**	0.05	−0.01	0.01	0.47	−0.52
	3. I feel like I have no one to share with.	**0.79**	−0.07	0.13	0.14	0.72	−0.26
	4. I feel lonely, even though I’m surrounded by people.	**0.65**	0.08	0.07	0.21	0.61	−0.80
	5. I feel like no one is interested in me.	**0.65**	0.31	0.00	0.03	0.66	−0.28
	6. I feel like no one values me.	**0.54**	0.50	0.03	−0.05	0.70	−0.17
	7. I feel like no one understands me.	**0.51**	0.45	0.03	−0.01	0.91	0.72
	8. I feel like I cannot find a purpose for my life.	**0.50**	0.29	−0.06	0.18	1.54	2.07
Factor 2: Family Estrangement							
	9. I feel like my family does not value me.	−0.06	**0.84**	0.17	−0.04	0.66	−0.39
	10. I feel like my family does not understand me.	0.04	**0.76**	0.05	0.03	1.04	0.40
	11. My family is too busy with their own things to pay attention to me.	−0.03	**0.70**	0.00	0.20	1.15	0.94
	12. I feel like I’m not a part of my family.	0.19	**0.64**	−0.01	0.04	1.98	3.74
	13. I feel separated from my family.	0.09	**0.62**	−0.07	0.20	1.07	1.05
	14. It is difficult for me to talk about personal topics with my family.	0.08	**0.54**	−0.04	0.19	1.32	1.48
Factor 3: Loss of Attachment Figure							
	15. I think about an *important person to me* who is no longer with me.	−0.01	0.01	**0.95**	−0.10	0.96	0.73
	16. I recall moments I shared with a person who is no longer with me.	−0.06	0.03	**0.92**	0.00	1.33	1.63
	17. I see photos or videos of an *important person to me* who is no longer with me.	0.08	−0.06	**0.89**	−0.02	1.63	2.31
	18. I have strong desires for an *important person to me*, to be by my side again.	0.08	0.09	**0.77**	0.05	1.05	0.75
	19. I have feelings that I wish to express to a person who is no longer with me.	−0.02	−0.04	**0.71**	0.23	1.06	0.52
	20. I feel discomfort being distant from an important person in my life.	0.01	0.14	**0.55**	0.23	0.77	−0.06
Factor 4: Intrapersonal Emptiness							
	21. I keep myself busy with various activities to avoid feeling lonely.	−0.01	0.07	−0.09	**0.85**	0.66	−0.21
	22. I dedicate several hours to my work to avoid feeling lonely.	0.08	−0.01	0.01	**0.82**	1.06	0.31
	23. I hang out with my friends to avoid feeling lonely.	0.02	−0.08	0.14	**0.72**	0.62	−0.66
	24. I play music or turn on the TV to avoid feeling lonely.	0.01	0.08	0.11	**0.68**	1.06	0.60
	25. I spend a lot of time on social media to avoid feeling lonely.	0.18	0.14	0.15	**0.53**	1.46	1.37

Note. *N* = 242. *s* = skewness; *k* = kurtosis. Factor loadings above 0.40 are in bold.

**Table 5:** Results of confirmatory factor analysis for the Multidimensional Loneliness Scale.

Model	χ2	*df*	CFI	RMSEA	SRMR	AIC
A: Unidimensional Model ^a^	1630.4 **	275	0.67	0.14	0.11	1730.41
B: Four-Dimensional Model ^b^	625.8 **	269	0.91	0.07	0.07	737.87

Note. *N* = 242. χ2 = chi-square value of model fit; df = degree of freedom; CFI = comparative fit index; RMSEA = root mean square error of approximation; SRMR = standardized root mean square residual; AIC = Akaike information criterion.; ^a^ In Model A, the 25 items loaded onto a single factor. ^b^ In Model B, the 8 items for social disconnection loaded onto one factor, the 6 items for family estrangement loaded onto a second factor, the 6 items for loss of attachment figure loaded onto a third factor, and the 5 items for intrapersonal emptiness loaded onto a fourth factor.; ** *p* < 0.01.

**Table 6:** Concurrent validity.

Scale	Multidimensional Loneliness Scale (MLS)			
	SOD	FAE	LAF	INE
1. De Jong Gierveld Loneliness Scale	0.65 **	0.59 **	0.31 **	0.52 **
1.1. Emotional Loneliness	0.44 **	0.40 **	0.14 *	0.23 **
1.2. Social Loneliness	0.65 **	0.59 **	0.38 **	0.62 **
2. Revised UCLA Loneliness Scale	0.75 **	0.61 **	0.20 **	0.52 **

Note. *N* = 242. SOD = social disconnection; FAE = family estrangement; LAF = loss of attachment figure; INE = intrapersonal emptiness. * *p* < 0.05. ** *p* < 0.01.

**Table 7:** Results of reliability.

Variables		ω	α
Multidimensional Loneliness Scale (MLS)			
	Factor 1: Social Disconnection	0.93	0.93
	Factor 2: Family Estrangement	0.89	0.89
	Factor 3: Loss of Attachment Figure	0.89	0.90
	Factor 4: Intrapersonal Emptiness	0.84	0.84

Note. *N* = 242. ω = coefficient omega; α = coefficient alpha.

**Table 8:** Conversion of raw scores to percentiles.

Percentile	Social Disconnection	Family Estrangement	Loss of Attachment Figure	Intrapersonal Emptiness
5	8	6	6	5
10	8	6	8	5
15	8	6	9	6
20	9	7	10	6
25	9	7	10	7
30	10	8	11	7
35	11	8	11	8
40	11	9	12	9
45	12	9	12	9
50	13	10	12	10
55	14	10	13	10
60	15	11	13	10
65	15	12	14	11
70	16	12	15	12
75	17	13	16	13
80	18	14	17	13
85	20	14	19	14
90	21	16	20	15
95	25	18	22	16

Note. Percentiles were calculated empirically based on the observed raw score distributions in the sample. Some percentiles share the same raw score due to the discrete nature of the data and sample size.

## Data Availability

The data that support the findings of this study are available from the corresponding author upon reasonable request.
